# Hypoxia‐inducible factor 1 promotes chemoresistance of lung cancer by inducing carbonic anhydrase IX expression

**DOI:** 10.1002/cam4.991

**Published:** 2016-12-28

**Authors:** Terumasa Sowa, Toshi Menju, Toyofumi F. Chen‐Yoshikawa, Koji Takahashi, Shigeto Nishikawa, Takao Nakanishi, Kei Shikuma, Hideki Motoyama, Kyoko Hijiya, Akihiro Aoyama, Toshihiko Sato, Makoto Sonobe, Hiroshi Harada, Hiroshi Date

**Affiliations:** ^1^ Department of Thoracic SurgeryGraduate School of MedicineKyoto University54 Kawaharacho, ShogoinSakyo‐kuKyoto606‐8507Japan; ^2^ Laboratory of Cancer Cell BiologyRadiation Biology CenterKyoto UniversityYoshida KonoechoSakyo‐kuKyoto606‐8501Japan; ^3^ Precursory Research for Embryonic Science and Technology (PRESTO)Japan Science and Technology Agency (JST)4‐1‐8 HonchoSaitama332‐0012Japan

**Keywords:** Carbonic anhydrase IX, chemoresistance, hypoxia‐inducible factor 1, induction chemoradiotherapy, lung cancer

## Abstract

Lung cancer treatment is difficult owing to chemoresistance. Hypoxia‐inducible factor 1 (HIF‐1) and HIF‐1‐induced glycolysis are correlated with chemoresistance; however, this is not evident in lung cancer. We investigated the effect of HIF‐1*α* and carbonic anhydrase IX (CAIX), a transmembrane protein neutralizing intracellular acidosis, on chemoresistance and prognosis of lung cancer patients after induction chemoradiotherapy. Associations of HIF‐1*α*, glucose transporter 1 (GLUT1), and CAIX with chemoresistance of lung cancer were investigated using A549 lung cancer cells under normoxia or hypoxia in vitro. HIF‐1*α*‐induced reprogramming of glucose metabolic pathway in A549 cells and the effects of HIF‐1 and CAIX on the cytotoxicity of vinorelbine were investigated. Immunohistochemical analyses were performed to determine HIF‐1*α*, GLUT1, and CAIX expression levels in cancer specimens from lung cancer patients after induction chemoradiotherapy. Hypoxia induced HIF‐1*α* expression in A549 cells. Moreover, hypoxia induced GLUT1 and CAIX expression in A549 cells in a HIF‐1‐dependent manner. Glucose metabolic pathway was shifted from oxidative phosphorylation to glycolysis by inducing HIF‐1*α* in A549 cells. HIF‐1 and CAIX induced chemoresistance under hypoxia, and their inhibition restored the chemosensitivity of A549 cells. The expression levels of HIF‐1*α*, GLUT1, and CAIX were associated with poor overall survival of lung cancer patients after induction chemoradiotherapy. HIF‐1 and CAIX affected the chemosensitivity of A549 cells and prognosis of lung cancer patients. Therefore, inhibition of HIF‐1 and CAIX might improve prognosis of lung cancer patients after induction chemoradiotherapy. Further analysis might be helpful in developing therapies for lung cancer.

## Introduction

Lung cancer is the leading cause of cancer deaths worldwide 1. Recently, various therapeutic strategies for lung cancer have been developed; however, these strategies are not sufficient for overcoming lung cancer. Patients with early‐stage lung cancer are usually treated by surgical resection, which is successful in most cases. On the other hand, patients with advanced lung cancer are often treated using multimodal therapy, including chemotherapy, radiotherapy, and surgical resection. Some studies have provided promising results for the effects of multimodal therapy; however, this therapeutic effectiveness has not been completely evident 2. Chemoresistance is an important limitation that hampers the improvement in prognosis of lung cancer patients.

Our previous studies have shown that prognosis of patients with advanced nonsmall cell lung cancer (NSCLC), especially stage IIIA NSCLC patients with mediastinal lymph node metastasis, can be improved using multimodal therapy 3. However, cancer progression during chemotherapy or relapse after postoperative therapy often occurs probably because of the induction of chemoresistance through some mechanisms.

Hypoxic microenvironment in a malignant solid tumor has been reported to be strongly correlated with cancer progression and chemoresistance. Hypoxia‐inducible factor 1 (HIF‐1), which is a heterodimeric transcription factor composed of *α* and *β* subunit (HIF‐1*α* and HIF‐1*β*, respectively) and whose activity is mainly dependent on the expression levels of the former, is recognized as a key factor associated with chemoresistance of lung cancer 4, with HIF‐1‐induced glycolysis playing an important role in promoting this chemoresistance 5.

HIF‐1‐induced glycolysis exerts many effects on tumor progression and on the mode of energy production 5. A recent study showed associations between glycolysis and chemoresistance 6. Upregulation of glycolysis increases glucose uptake through glucose transporter 1 (GLUT1) 5, a key regulator of glycolysis that has been investigated as a diagnostic tool and therapeutic target for cancer 7.

Because intracellular acidosis is induced by lactate and proton production during glycolysis, the mechanism coping with intracellular acidosis is essential for the maintenance of cellular homeostasis. As a coping mechanism, carbonic anhydrase IX (CAIX), a transmembrane protein neutralizing intracellular acidosis, is induced by HIF‐1 and is associated with glycolysis in some cancers 8. Some studies have reported the effects of CAIX on prognosis and chemoresistance of patients with different cancers 9. However, few studies have reported the effects of CAIX on prognosis and chemoresistance of lung cancer patients.

HIF‐1‐induced glycolysis is highly associated with cancer progression. Therefore, the investigation of this pathway in lung cancer is important for developing novel cancer therapies. The purpose of our study is to elucidate the effects of HIF‐1*α* and CAIX on chemoresistance and prognosis of lung cancer patients after induction chemoradiotherapy, and finally to improve them.

## Materials and Methods

### Cell culture and reagents

Human lung adenocarcinoma cell‐line A549 was obtained from American Type Culture Collection (Manassas, VA) and was cultured in Dulbecco's modified Eagle's medium (DMEM; Sigma‐Aldrich, St. Louis, MO) containing 10% fetal bovine serum (FBS).

For normoxic experiments, A549 cells were incubated in a well‐humidified incubator with 5% CO_2_ and 95% air at 37°C. For hypoxic experiments, A549 cells were incubated with <0.1% O_2_ in Bactron Anaerobic Chamber, BACLITE‐2 (Sheldon Manufacturing, Cornelius, OR).

HIF‐1 inhibitor YC‐1 (Cayman Chemical Company, Ann Arbor, MI) was dissolved in dimethyl sulfoxide at a concentration of 20 mg/mL to prepare a stock solution. Vinorelbine was purchased from WAKO (Osaka, Japan).

### Plasmids and transfection

A549 cells were trypsinized, counted, and seeded 1 day before transfection to achieve 70% confluency on the day of making lysates.

Small‐interfering RNAs (siRNAs) targeting *HIF‐1α* were purchased from Invitrogen (Waltham, MA), and siRNAs targeting *CAIX* and control siRNAs were purchased from Japan Bio Services Co., LTD (Saitama, Japan). A549 cells were transfected with the specific siRNAs (final concentration, 120 nmol/L) by using RNAiMAX (Invitrogen), according to the manufacturer's instructions. Targeting sequences of *HIF‐1α*,* CAIX*, and control siRNAs are as follows:

1. *HIF‐1α*
  5ʹ‐GAAAUUCCUUUAGAUAGCAAGACUU‐3ʹ
  5ʹ‐AGUUAGUUCAAACUGAGUUAAUCCC‐3ʹ
2. *CAIX*
  5ʹ‐GCAACAAUGGCCACAGUGU‐3ʹ
  5ʹ‐GGAAGAAAUCGCUGAGGAA‐3ʹ
3. Nonspecific control siRNAs (negative control)
  5ʹ‐GCGCGCUUUGUAGGAUUCG‐3ʹ

The plasmid expressing a short hairpin RNA (shRNA) targeting *HIF‐1α* was purchased from Qiagen (Hilden, Germany), and stably transfected into A549 cells through the calcium phosphate method 10. The resultant cells were cultured in selection medium containing puromycin (1 *μ*g/mL) for 14 days to establish *HIF‐1α*‐silencing A549 cells.

To construct pcDNA4/HIF‐1*α* 3A, which expresses a constitutively active mutant HIF‐1*α*, pcDNA4/HIF‐1*α* plasmid was introduced with three‐point mutations, P402A, P564A, and N803A (HIF‐1*α* 3A herein), through the site‐directed mutagenesis. The mutations in proline and asparagine residues enable HIF‐1*α* protein to avoid the prolyl 4‐hydroxylases (PHDs)‐mediated proteolysis and the factor‐inhibiting HIF‐1‐dependent suppression of transactivation activity of HIF‐1*α*, respectively 11. Cells were transfected with this recombinant vector or control vector by using Lipofectamine 2000 (Invitrogen), according to the manufacturer's instructions.

### Western blotting

A549 cells were seeded 1 day before treatment to ensure 70% cell confluency on the day of making lysates. The cells were treated with the reagents or the vectors for 24 h. Cell lysates were harvested by lysing the cells in 4‐(2‐hydroxyethyl)‐1‐piperazineethanesulfonic acid buffer containing protease inhibitors (1 mmol/L phenylmethylsulfonyl fluoride, 1 *μ*g/mL aprotinin, 1 *μ*g/mL pepstatin, 0.5 *μ*g/mL leupeptin, and 1 mmol/L vanadate). The lysates were immunoblotted using antibodies against HIF‐1*α* (610958, BD Bioscience, San Jose, CA), GLUT1 (ab40084, Abcam, Cambridge, UK), CAIX (M75, Bioscience Slovakia, Bratislava, Slovakia), and *β*‐actin (A5441, Sigma‐Aldrich), which was used as a control. For hypoxic experiments, cell lysates were harvested in a hypoxic chamber and were treated as described above.

### Cell proliferation assay

A549 cells were seeded in 96‐multiwell plates (3000 cells/well) containing DMEM supplemented with 10% FBS and were incubated overnight. The cells were then treated with vinorelbine and were incubated under normoxia or hypoxia for 72 h. Cell proliferation rate was measured using 4‐[3‐(4‐iodophenyl)‐2‐(4‐nitrophenyl)‐2H‐5‐tetrazolio]‐1,3‐benzenedisulfonate (WST‐1) colorimetric assay (Dojindo, Kumamoto, Japan), according to the manufacturer's instructions. All measurements were performed in triplicate.

### Metabolic flux assay

Bioenergetic flux of HIF‐1*α*‐overexpressing cells under normoxia was assessed using Seahorse XF96 extracellular flux analyzer (Seahorse Biosciences, North Billerica, MA). For this, A549 cells, which were transfected with the vector expressing the constitutively active mutant HIF‐1*α* or control vector for 24 h, were seeded in XF96 plates at 50,000 cells/well (after optimization of cell seeding number) and were incubated with DMEM in 5% CO_2_ at 37°C for 24 h. Cell culture medium was replaced with XF medium (Seahorse Biosciences) containing 10% glutamine and lacking sodium bicarbonate and FBS. The cells were then incubated in the absence of CO_2_ at 37°C for 1 h before initiating the experiment. Basal extracellular acidification rate (ECAR), which indicates proton leakage during glycolysis, and basal oxygen consumption rate (OCR), which indicates mitochondrial respiration, were measured using XF96 plate reader according to the manufacture's instruction. OCR and ECAR values were normalized using cell counts (ECAR: mpH/[min · 10^4^ cells], OCR: pmol/[min · 10^4^ cells]).

### Immunohistochemical analysis

Immunohistochemical analysis of HIF‐1*α*, GLUT1, and CAIX was performed using a standard technique published previously 12. Briefly, HIF‐1*α*, GLUT1, and CAIX were immunostained using rabbit anti‐human HIF‐1*α* polyclonal antibody (dilution, 1:500; Novus, Littleton, CO), mouse anti‐human GLUT1 monoclonal antibody (ab40084; dilution, 1:200), and mouse anti‐human CAIX monoclonal antibody (M75, 1:100), respectively. Next, ABC method was performed using Vectastain ABC kit (Tokyo, Japan), according to the manufacturer's instructions.

Immunostained sections were examined by two authors (T. S. and T. M.) who were blinded to patient characteristics. Cases showing discrepancies were jointly reevaluated until consensus was reached. HIF‐1*α*, GLUT1, and CAIX expression was examined in four distinct fields containing a minimum of 500 cells. The proportion of positive cells was measured and classified as 0 (no staining), +1 (weak), +2 (moderate), and +3 (strong), and each specimen was categorized as negative (0 and +1) or positive (+2 and +3). Representative images of immunohistochemical staining are shown in Figure 4A.

For this analysis, in all 25 NSCLC specimens were collected from stage IIIA advanced NSCLC patients with pathologically proven mediastinal lymph node metastasis (N2) who received induction chemoradiotherapy at Kyoto University Hospital from 2006 to 2014. These patients were treated by platinum‐doublet chemotherapy (carboplatin and paclitaxel or cisplatin and vinorelbine) with concurrent radiotherapy. Tumors were staged using the 7th Edition of the TNM classification of the International Union Against Cancer 13. Survival time and outcome data were available for all the 25 patients. Median follow‐up duration of the 25 patients was 45 months (range: 14–111 months). Use of the patient specimens in this study was approved by the Institutional Review Board of the Kyoto University Hospital. Written informed consent was obtained from all of the patients.

### Statistical analysis

Differences in ECAR and OCR values were statistically analyzed using *t*‐test. Time‐to‐event curves for overall survival (OS) and disease‐free survival (DFS) were estimated using Kaplan–Meier method, and differences in these curves were calculated using log‐rank test. Correlations among the expression of HIF‐1*α*, GLUT1, and CAIX in the clinical specimens were analyzed using chi‐square test. All *P*‐values were two sided, and *P *<* *0.05 was considered statistically significant. These analyses were performed using JMP Pro 11 (SAS, Cary, NC). Best‐fit IC50 values were compared using an extra sum‐of‐square *F* test with Prism 6 (GraphPad Software Inc., San Diego, CA).

## Results

### In vitro study

#### Hypoxia induced HIF‐1*α*, GLUT1, and CAIX expression in lung cancer cells

First, we studied the behavior of HIF‐1*α*, GLUT1, and CAIX in the lung cancer cell‐line A549 under normoxia and hypoxia.

**Figure 1:**
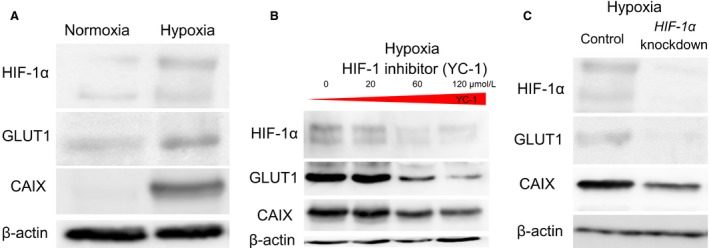
Hypoxia induced HIF‐1*α*, GLUT1, and CAIX expression in A549 cells. Moreover, hypoxia induced GLUT1 and CAIX expression in a HIF‐1‐dependent manner. (A) HIF‐1*α*, GLUT1, and CAIX expression in A549 cells was stronger under hypoxia than under normoxia. (B) HIF‐1 inhibitor YC‐1 suppressed HIF‐1*α*, GLUT1, and CAIX expression under hypoxia in a dose‐dependent manner. (C) *HIF‐1α* siRNAs suppressed GLUT1 and CAIX expression under hypoxia. It indicated that hypoxia induced GLUT1 and CAIX expression in A549 cells in a HIF‐1‐dependent manner. HIF‐1*α*, hypoxia‐inducible factor 1*α*; GLUT1, glucose transporter 1; CAIX, carbonic anhydrase IX.

A549 cells were incubated under normoxia or hypoxia for 24 h, and expression of HIF‐1*α*, GLUT1, and CAIX in cell lysates was determined by performing Western blotting. It was shown that HIF‐1*α*, GLUT1, and CAIX expression was stronger under hypoxia than under normoxia (Fig. 1A). Our results showed that hypoxia induced the expression of GLUT1, which is associated with the intake of glucose into cells, and CAIX, which neutralizes glycolysis‐associated intracellular acidosis, in A549 cells.

#### Hypoxia induced GLUT1 and CAIX expression in lung cancer cells in a HIF‐1‐dependent manner

Because our results showed that hypoxia induced GLUT1 and CAIX expression in A549 cells, we investigated whether this expression was induced in a HIF‐1‐dependent manner.

HIF‐1 inhibitor YC‐1 was used to inhibit HIF‐1*α* in A549 cells. A549 cells were seeded and incubated with YC‐1 (20, 60, and 120 *μ*mol/L) for 24 h. Results of Western blotting showed that inhibition of HIF‐1 with YC‐1 suppressed GLUT1 and CAIX expression in a dose‐dependent manner (Fig. 1B). The involvement of HIF‐1 was further confirmed by treating A549 cells with *HIF‐1α* siRNAs for 24 h. Our results showed that GLUT1 and CAIX expression was suppressed in *HIF‐1α*‐knockdown A549 cells (Fig. 1C).

These results indicated that hypoxia induced GLUT1 and CAIX expression in a HIF‐1‐dependent manner and that HIF‐1 was a key regulator of GLUT1 and CAIX expression in lung cancer cells.

#### HIF‐1 induced glycolysis in lung cancer cells

Above results suggested a possibility that HIF‐1‐dependent induction of GLUT1 expression led to the increase in glucose consumption. However, it was unclear whether upregulation of GLUT1 expression and increase in glucose consumption were induced by glycolysis rather than by oxidative phosphorylation.

Therefore, we used the extracellular flux analyzer to investigate whether HIF‐1 induced glycolysis. We measured basal ECAR, an index of glycolysis, and basal OCR, an index of oxidative phosphorylation, in A549 cells with or without genetical activation of HIF‐1*α* under normoxia. Because experiments involving the extracellular flux analyzer are performed under normoxia, we overexpressed HIF‐1*α* in A549 cells under normoxia by transfecting these cells with the vector encoding the constitutively active mutant HIF‐1*α* with three mutations, P402A, P564A, and N803A (HIF‐1*α* 3A). The use of this mutant allowed the evaluation of direct effects of HIF‐1*α* even under normoxia.

**Figure 2:**
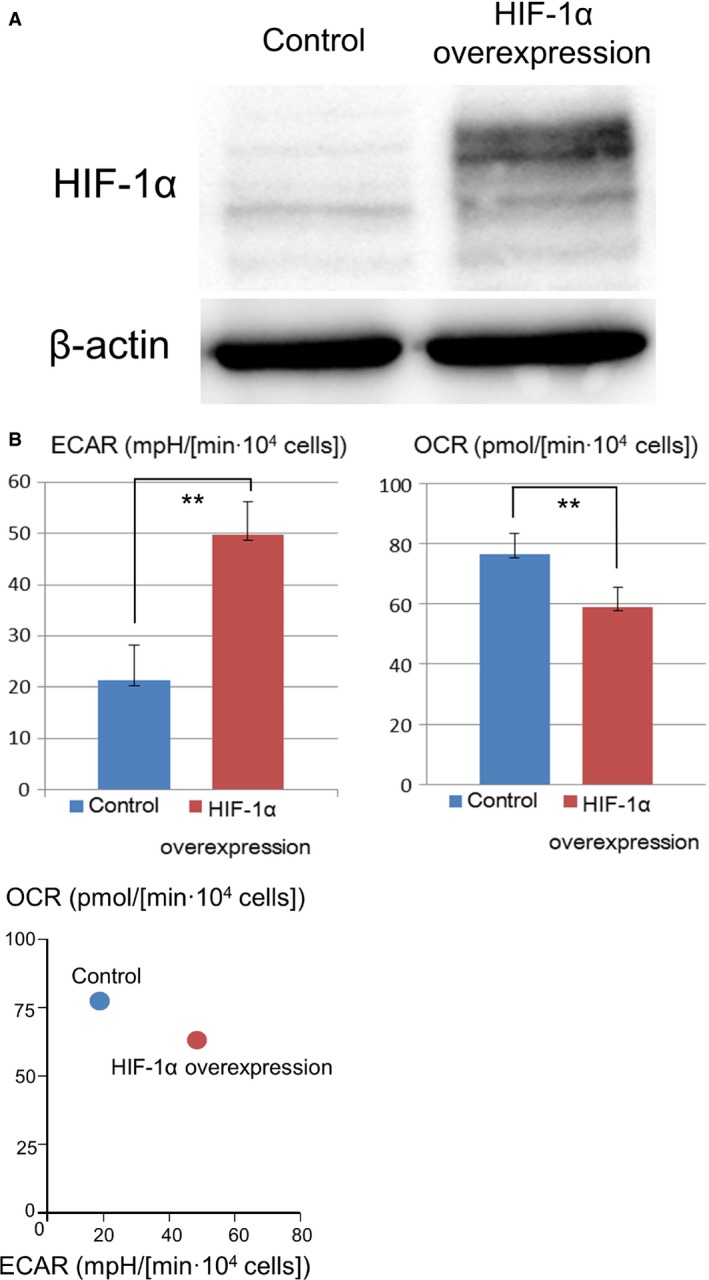
HIF‐1 induced glycolysis in A549 cells. (A) Western blotting detected HIF‐1*α* overexpression in A549 cells transfected with the vector encoding the constitutively active mutant HIF‐1*α*. (B) Under normoxia, ECAR increased by 133% and OCR decreased by 23.7% in HIF‐1*α* 3A‐overexpressing A549 cells compared with that in control cells (*t*‐test, **P <* *0.001). OCR and ECAR values were standardized using cell counts. Data are presented as mean ± SD (*n* = 12). HIF‐1, hypoxia‐inducible factor 1; ECAR, extracellular acidification rate; OCR, oxygen consumption rate.

Western blotting detected HIF‐1*α* overexpression in A549 cells transfected with the vector encoding the constitutively active mutant HIF‐1*α* (Fig. 2A), hereinafter referred to as HIF‐1*α* 3A‐overexpressing A549 cells. Experiment using the extracellular flux analyzer showed that the ECAR increased significantly (by 133%) and OCR decreased significantly (by 23.7%) in HIF‐1*α* 3A‐overexpressing A549 cells compared with that in control cells (Fig. 2B).

These results indicated that HIF‐1 shifted glucose metabolic pathway from oxidative phosphorylation to glycolysis in A549 cells.

#### HIF‐1 induced chemoresistance of lung cancer cells to vinorelbine

**Figure 3:**
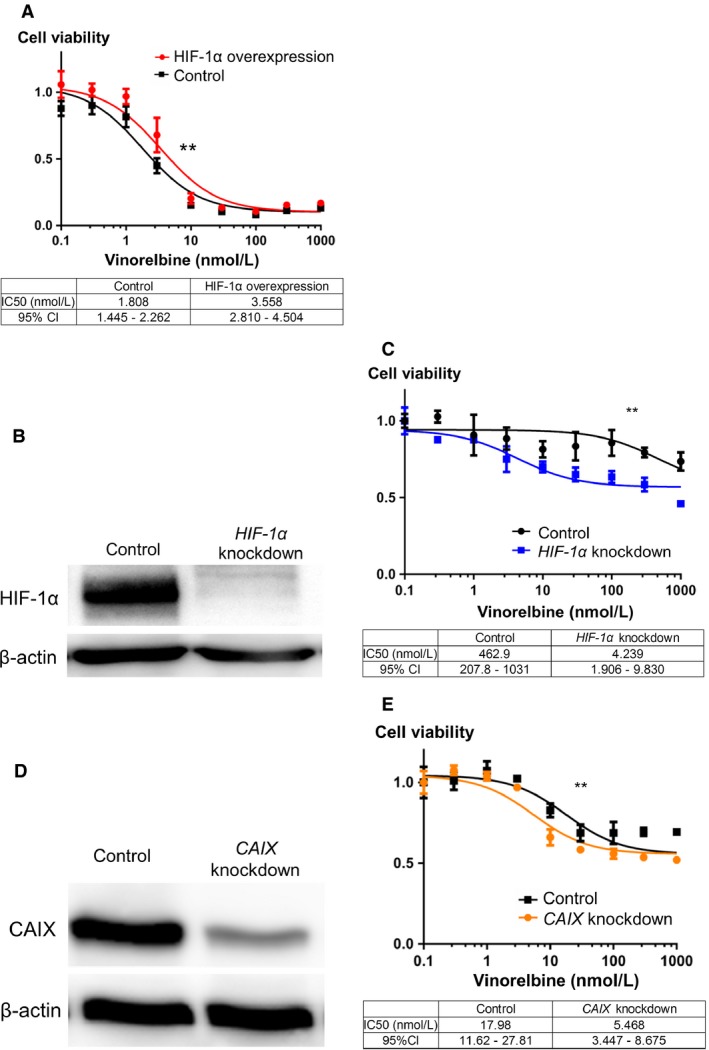
HIF‐1 and CAIX affected the chemosensitivity of A549 cells under hypoxia. (A) Under normoxia, HIF‐1*α* 3A‐overexpressing A549 cells showed significant chemoresistance to vinorelbine in WST‐1 assay. (B) Stable *HIF‐1α*‐knockdown A549 cells, which were obtained by treatment with *HIF‐1α* shRNA vector, showed suppressed HIF‐1*α* expression compared with control cells. (C) Under hypoxia, *HIF‐1α*‐knockdown A549 cells showed more chemosensitivity to vinorelbine than control cells in WST‐1 assay. (D) *CAIX*‐knockdown A549 cells, which were transfected with *CAIX* siRNAs, showed suppressed CAIX expression compared with control cells. (E) Under hypoxia, *CAIX*‐knockdown cells showed more chemosensitivity to vinorelbine than control cells in WET‐1 assay. Best‐fit IC50 values were compared using the extra sum‐of‐square *F* test by using Prism 6 (**P <* *0.001). Each value was standardized using that obtained for control cells. HIF‐1, hypoxia‐inducible factor 1; CAIX, carbonic anhydrase IX; WST1, 4‐[3‐(4‐iodophenyl)‐2‐(4‐nitrophenyl)‐2H‐5‐tetrazolio]‐1,3‐benzenedisulfonate.

Our results showed that HIF‐1 induced glycolysis and CAIX expression in A549 cells. Therefore, we examined the effects of HIF‐1*α* expression on chemoresistance of lung cancer. Cytotoxicity was measured by performing WST‐1 assay with or without HIF‐1*α* overexpression under normoxia. HIF‐1*α* overexpression was induced using the vector encoding the constitutively active mutant HIF‐1*α* (Fig. 2A). Under normoxia, the HIF‐1*α* 3A‐overexpressing A549 cells showed significant chemoresistance to vinorelbine (Fig. 3A), suggesting that HIF‐1 induced chemoresistance of lung cancer.

#### HIF‐1 inhibition under hypoxia restored the chemosensitivity of lung cancer cells to vinorelbine

Next, we investigated whether HIF‐1 inhibition restored the chemosensitivity of A549 cells to vinorelbine under hypoxia. For this, we established stable *HIF‐1α*‐knockdown A549 cells by silencing *HIF‐1α* expression with a shRNA expression vector (Fig. 3B). We compared the chemoresistance of *HIF‐1α*‐knockdown and control A549 cells to vinorelbine by WST‐1 assay.

Our results showed that *HIF‐1α*‐knockdown A549 cells were significantly more chemosensitive to vinorelbine than control cells under hypoxia (Fig. 3C), suggesting that HIF‐1 inhibition restored the chemosensitivity of A549 cells to vinorelbine under hypoxia.

#### CAIX inhibition under hypoxia restored the chemosensitivity of lung cancer cells to vinorelbine

In our study, we found that HIF‐1 induced glycolysis, CAIX expression, and chemoresistance in A549 cells. Therefore, we hypothesized that HIF‐1‐induced chemoresistance was mediated by CAIX expression. We investigated whether CAIX inhibition under hypoxia affected the chemosensitivity of A549 cells. Cytotoxic effects of vinorelbine on *CAIX*‐knockdown A549 cells, which were obtained by transfecting A549 cells with *CAIX* siRNAs (Fig. 3D), under hypoxia was measured by performing the WST‐1 assay.

Our results showed that *CAIX*‐knockdown cells were significantly more chemosensitive to vinorelbine than control cells under hypoxia (Fig. 3E), suggesting that CAIX inhibition under hypoxia restored the chemosensitivity of A549 cells.

### Clinical study

#### Clinical significance of HIF‐1*α* and other hypoxia‐associated metabolic factors in prognosis of NSCLC patients after induction chemoradiotherapy

**Figure 4:**
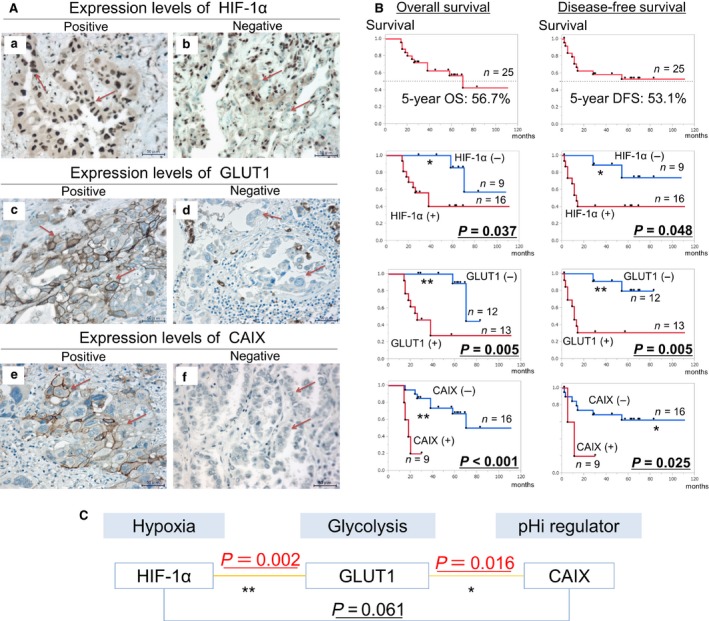
Analysis of the associations between HIF‐1*α*, GLUT1, and CAIX expression and prognosis of NSCLC patients after induction chemoradiotherapy. (A) Representative images of the immunohistochemical staining of NSCLC sections with anti‐HIF‐1*α* (a and b), GLUT1 (c and d), and CAIX (e and f) antibodies. (a, c, and e) show positive expression, whereas (b, d, and f) show negative expression. (Scale bar, 50 *μ*m, Original magnification, 400×.) (B) Results of Kaplan–Meier method and log‐rank test showed that HIF‐1*α*, GLUT1, and CAIX expression was significantly associated with poor OS and DFS of NSCLC patients who received induction chemoradiotherapy (OS: HIF‐1*α*,* P* = 0.037; GLUT1, *P* = 0.005; CAIX,* P* < 0.001; DFS: HIF‐1*α*,* P* = 0.048; GLUT1, *P* = 0.005; CAIX,* P* = 0.025). *P <* *0.05, **P <* *0.01. (C) Mutual association was observed between the expression of HIF‐1 and GLUT1 and between the expression of GLUT1 and CAIX (Chi‐squared test: HIF‐1*α*–GLUT1, *P *=* *0.002; GLUT1–CAIX,* P *=* *0.016), but not between the expression of HIF‐1*α* and CAIX (*P *=* *0.061). *P <* *0.05, **P <* *0.01. HIF‐1*α*, hypoxia‐inducible factor 1*α*; GLUT1, glucose transporter 1; CAIX, carbonic anhydrase IX; NSCLC, nonsmall cell lung cancer; OS, overall survival; DFS, disease‐free survival.

We investigated the effects of hypoxia‐associated factors on prognosis of NSCLC patients after induction chemoradiotherapy and surgery. For this, resected specimens from 25 patients of stage IIIA NSCLC who received induction chemoradiotherapy were collected at Kyoto University Hospital from 2006 to 2014. HIF‐1*α*, GLUT1, and CAIX expression in the collected specimens were examined by performing immunohistochemical analyses (Fig. 4A). Associations between the expression of these factors and patient prognosis were statistically analyzed. Clinicopathological characteristics of the patients evaluated in our study are summarized in Table 1.

**Table 1:** Characteristics of the patients included in this study

Variables	*N* = 25
Gender, *n* (%)	
Male	18 (72%)
Female	7 (28%)
Age (years)	61.5 ± 6.9 years old
Follow‐up periods (median)	45 months (14–111 months)
Pathological type	
Adenocarcinoma	15 (60%)
Squamous carcinoma	10 (40%)
Operative procedure	
Lobectomy	22 (88%)
Bilobectomy	2 (8%)
Pneumonectomy	1 (4%)
Pathological effect grade	
1	5 (20%)
2	14 (56%)
3	6 (24%)

Results of Kaplan–Meier method and log‐rank test showed that HIF‐1*α*, GLUT1, and CAIX expression was significantly associated with poor OS and DFS of NSCLC patients who received induction chemoradiotherapy (OS: HIF‐1*α*,* P *=* *0.037; GLUT1, *P *=* *0.005; CAIX, *P *<* *0.001; DFS: HIF‐1*α*,* P *=* *0.048; GLUT1, *P *=* *0.005; CAIX, *P *=* *0.025) (Fig. 4B). Mutual associations were significantly observed between the expression of HIF‐1*α* and GLUT1 and between the expression of GLUT1 and CAIX. (Chi‐squared test: HIF‐1*α*‐GLUT1, *P *=* *0.002; GLUT1‐CAIX, *P *=* *0.016) but not between the expression of HIF‐1*α* and CAIX (*P *=* *0.061) (Fig. 4C). These results indicated that expression of HIF‐1*α*, GLUT1, and CAIX was correlated with poor prognosis of NSCLC patients after induction chemoradiotherapy.

## Discussion

In this study, we found that HIF‐1 and its associated factor CAIX induced chemoresistance of A549 cells and that these factors were associated with prognosis of stage IIIA advanced NSCLC patients after induction chemoradiotherapy. To our knowledge, this is the first study to report these results for lung cancer. Moreover, these results may help in developing novel strategies for treating advanced lung cancer.

Previous studies have reported that HIF‐1 plays important roles in cancer progression and metastasis 4. Activity of HIF‐1 is mainly regulated in an oxygen‐dependent manner. PHDs hydroxylates the proline residues in the oxygen‐dependent degradation domain of HIF‐1*α* under normoxia. This hydroxylation triggers the ubiquitination of HIF‐1*α* by VHL‐containing E3 ubiquitin ligase, leading to its proteolysis 14, 15. HIF‐1 activity is mainly regulated by HIF‐1*α* in an oxygen‐dependent manner, and it indicates that HIF‐1*α* is one of the most important factors induced under hypoxia.

HIF‐1*α* has been reported to promote cancer progression through multiple mechanisms 4. Moreover, clinical studies have shown that HIF‐1*α* promotes cancer metastasis and relapse 16. Our previous study showed that HIF‐1*α* and ubiquitin C‐terminal hydrolase‐L1 were correlated with cancer metastasis and were associated with prognosis of patients with lung adenocarcinoma 11. In our study, we could provide new insights on HIF‐1*α* in lung cancer.

Although various mechanisms underlying the effect of HIF‐1 on cancer progression have been reported, clinical application of HIF‐1 inhibitors for inhibiting cancer progression has not been performed to date 17. HIF‐1 exists in the nuclei of cancer cells; therefore, HIF‐1 inhibitors may not be able to reach it. In contrast, CAIX is a cell surface protein that can be easily accessed for treatment and is almost exclusively expressed on cancer cells but not on surrounding normal cells, except gastric cells. This is one of the reasons why we focused on CAIX in our study.

CAIX, an intracellular pH regulator that induces tissue growth and development, is associated with hypoxia and glycolysis. CAIX neutralizes intracellular acidosis induced by glycolysis‐associated lactate and proton along with a bicarbonate transporter 8, 18. In our study, we found that CAIX induced chemoresistance of A549 cells. Although we did not prove a causal relationship between CAIX expression and chemoresistance, the two mechanisms were mainly reported 9, 18, 19, 20. First, chemotherapy induces intracellular acidosis, and CAIX prevents apoptosis by maintaining a normal intracellular pH in response to chemotherapy 9. Second, CAIX‐induced extracellular acidosis is responsible for chemoresistance of cancer cells. CAIX converts CO_2_ and H_2_O into H^+^ and HCO_3_^−^ in the extracellular space, which induces intracellular alkalosis through bicarbonate transporter and H^+^‐induced extracellular acidosis in the extracellular space. Most anticancer drugs, including vinorelbine, are charged weak bases and become protonated and impaired at acidic extracellular pH 18, 19, 20. Thus, CAIX inhibition may suppress chemoresistance of cancer cells through these two mechanisms.

Our clinical analysis showed that HIF‐1*α* and CAIX were associated with prognosis of NSCLC patients after induction chemoradiotherapy. Although some studies have reported the importance of CAIX in prognosis of lung cancer patients 21, the exact mechanism remains unclear. On the other hand, our analysis of 239 resected lung adenocarcinoma specimens at our institute did not show the association of CAIX expression with patient prognosis (data not shown) 22. These results may provide new insights on using CAIX as a target for treating advanced lung cancer requiring induction chemoradiotherapy. Furthermore, clinical trials on novel inhibitors of CAIX are in progress 8, 23, and results of these trials are expected to improve patient prognosis. Thus, our study suggested that cancer therapy involving CAIX inhibition would provide promising results.

However, our study has limitations. First, data of only 25 patients with stage IIIA advanced NSCLC were included in our study. We selected the cases from a homogeneous group of patients limited to stage IIIA advanced NSCLC with pathologically proven mediastinal lymph node metastasis, for the sake of the accurate analysis. Future studies should be performed using data from more number of patients. Second, our results showed the association between HIF‐1‐associated factors and prognosis of lung cancer patients. However, they did not indicate whether these factors were predictors of the effectiveness of preoperative induction chemoradiotherapy. For the further investigation, 18F‐Fluoromisonidazole (FMISO) positron emission tomography is now in clinical trial in Kyoto University. FMISO accumulates in hypoxic regions of cancer tissues and allows their visualization 24, and it will determine whether hypoxic regions exist in lung cancer before the initiation of induction chemoradiotherapy and whether these regions can predict the effectiveness of induction chemoradiotherapy. It will also be useful for developing clinical therapies involving HIF‐1 and CAIX inhibitors, especially for treating NSCLC patients after induction chemoradiotherapy.

## Conclusion

Our results showed that HIF‐1 induced glycolysis and the expression of CAIX, which affected the chemosensitivity and prognosis of lung cancer patients. HIF‐1 and CAIX inhibition may improve patient prognosis, especially prognosis of stage IIIA NSCLC patients after induction chemoradiotherapy. Further studies on the novel applications of these factors would help in developing cancer therapies in the future.

## Conflict of Interest

None declared.
